# Sociodemographic characteristics and health status of women with breast cancer and COVID 19 diagnosis by menopausal status a cross sectional study

**DOI:** 10.1038/s41598-025-86710-8

**Published:** 2025-01-21

**Authors:** Mohammadhossein Hajiebrahimi, Hussam Shihan, Ola Bratt, Huiqi Li, Fredrik Nyberg, Björn Wettermark

**Affiliations:** 1 Department of Pharmacy, Faculty of Pharmacy, Uppsala University, Uppsala, Sweden; 2 Clincal Studies Department, University Hospital, Linköping, Region Östergötland, Sweden; 3 Department of Urology, Institute of Clinical Sciences, Sahlgrenska Academy, University of Gothenburg, Gothenburg, Sweden; 4 School of Public Health and Community Medicine, Institute of Medicine, Sahlgrenska Academy, University of Gothenburg, Gothenburg, Sweden; 5 Biomedicinskt Centrum BMC, Husargatan 3, 752 37 Uppsala, Sweden

**Keywords:** Breast cancer, COVID-19, Drug utilization, Health service use, Risk factors, Cancer, Breast cancer, Cancer epidemiology

## Abstract

The goal of this work is to investigate the sociodemographic characteristics and health status of women with breast cancer (BC) in association with COVID-19 by menopausal status. In a Swedish register-based cross-sectional study, we compared women with BC and with or without a positive COVID-19 test, stratified by menopausal status (age ≥ 51 years). Socioeconomic characteristics and health status (represented by diagnoses registered in 5 years- and prescription dispensed in 2 years preceding Jan 2020) were considered in association with COVID-19 diagnosis. The study population included 38,523 women with BC. Median age at BC diagnosis was 45 years (IQR = 40–48) for premenopausal- and 67 (IQR = 60–73) for postmenopausal BC. A logistic regression model was used and found the significant covariate effects (adjusted odds ratios, ORs) for a positive COVID-19 test among women with premenopausal BC to be being born outside of Europe: 1.29, (1.13–1.46), being married: 1.23, (1.12–1.36), being unemployed 1.92 (1.59–2.30), having upper secondary school education 1.25 (1.01–1.54), having > 15 outpatient visits: 1.31, (1.07–1.61), and a history of being admitted to hospital 1–5 times: 1.12 (1.01–1.25). Corresponding significant covariate effects among women with postmenopausal BC were being born outside of Europe: 1.61 (1.41–1.83), being married: 1.12 (1.04–1.21), and being unemployed 1.54 (1.40–1.69). Postmenopausal women furthermore had more outpatient visits or hospital admissions before the pandemic in COVID-19 positive patients compared to patients without a COVID-19 positive test, e.g. 1.47 (1.26–1.71) for > 15 outpatient visits compared with no visit and 6.35 (3.33–12.11) for > 15 hospital admissions compared with no admission. Varied socioeconomical and clinical conditions were more frequent among patients with a positive COVID-19 test compared to patients without a positive test among women with BC in pre- or post-menopausal status. We conclude that some characteristics of women such as unemployment, country of birth or health status measured by number of prescribed drugs were more prevalent among women who developed COVID-19 compared to women without COVID-19 diagnosis and either of menopausal status of breast cancer.

**Supplementary Information:**

The online version contains supplementary material available at 10.1038/s41598-025-86710-8.

## Introduction

Coronavirus Disease 2019 (COVID-19) was declared a pandemic between March 11, 2020^1^ and May 5, 2023^1^ and had a considerable impact on patients, health services and healthcare systems^2,3^. Underlying comorbidities including chronic diseases and cancers are known risk factors of diagnosis or severity of COVID-19. Although a meta-analysis has reported higher risk of COVID-19 infection and excess mortality among cancer patients^4^ and a systematic review protocol^5^ has aimed to study the association between breast cancer and COVID-19 severity, we found no study that investigated different types of cancer and specifically reported findings for women with pre- and postmenopausal breast cancer.

Breast cancer (BC) is the most commonly diagnosed cancer among women in the world^6^ and a leading cause of death among cancer patients globally^7^. In Sweden, breast cancer is the most frequent cancer and fifth leading cause of death among women^8^. Breast cancer, a heterogenous malignancy, is subdivided into two groups based on menopausal status: premenopausal and postmenopausal^9,10^. Premenopausal breast cancers constitute around 25% of all malignant breast tumors. They are characterized by lower expression of estrogen or/and progesterone receptors, and higher risk of mortality than postmenopausal breast cancers^11^. Owing to these differences, Breast Cancer studies commonly stratify results by menopausal status of women at the time of diagnosis.

Association between cancer and COVID-19 has been considered in different studies. A review study has reported that cancer apparently is a factor for poor prognosis of COVID-19^12^. A systematic review and meta-analysis concluded that cancer patients have a higher risk of severe COVID-19, increased need for ventilatory support, and higher mortality compared with the general population^13^. Another review, in contrast, reported that cancer cannot be considered as an independent risk factor for the prognosis of COVID-19. Poor prognosis was rather attributed to comorbidities and poor general status than to radiation therapy or current anti-cancer therapy of cancer patients^12^. Some recent studies investigated the severity or worse prognosis of COVID-19 among cancer patients including breast cancer. A cohort study of 928 cancer patients (including breast cancer) with data from a cancer database in the US, Canada and Spain reported that increased COVID-19 mortality was associated with several independent variables such as age, sex, number of comorbidities, and geographical area^14^. Consideration of breast cancer specifically has showed that breast cancer patients are more susceptible to COVID-19 than the general population^15,16^. A French study with 15,676 participants (76 cases, 15600 comparators)^17^ investigated outcomes of COVID-19 among breast cancer patients and concluded that assessment of comorbidities should be the first focus to define high-risk patients for COVID-19 among breast cancer patients. Shared pathological features between breast cancer and COVID-19, such as inflammatory mediators^15^, could be potential causes behind such susceptibility.

Despite investigations on the association between breast cancer and risk of or survival from COVID-19, we found no study that examined whether sociodemographic status could be associated with diagnosis of COVID-19 among breast cancer patients. Several published studies^18–24^ considered the association between socioeconomic status and risk or severity of COVID-19 among patients with cancer in general. Socioeconomic factors such as sex, education level, income, marital status, ethnicity, migration, employment status and health status, measured for example by health service use, as well as comorbidity, have been considered in these studies. The results have shown that the socioeconomic status of people with cancer is associated with diagnosis, severity and survival of COVID-19. A study from the United States^23^ has reported that the pattern of socioeconomic disparities seen in COVID-19 patients was consistent with the disparity patterns in cancer patients, suggesting common causes of disparity for COVID-19 and cancer. Thus, given the importance of the topic and the limited data specific to breast cancer patients, we aimed to investigate whether baseline sociodemographic characteristics, or health status as measured by history of health service uses and history of drug utilization, are associated with diagnosis of COVID-19 infection in women with breast cancer diagnosis. We separately studied premenopausal and postmenopausal women with breast cancer.

## Materials and methods

**Fig. 1:**
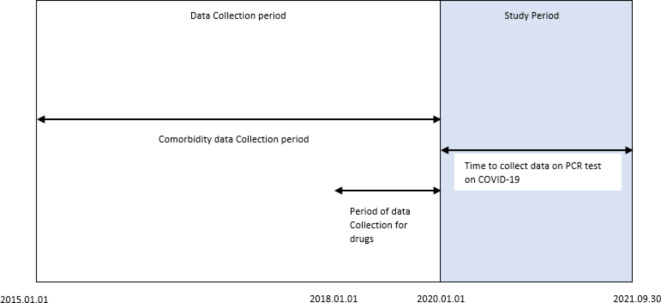
Time frame of the study period and the data collection period.

Using data from the Swedish COVID-19 Investigation for Future Insights – a Population Epidemiology Approach using Register Linkage (SCIFI-PEARL) project dataset including linked national health registers^25^, we designed a cross-sectional study. The population of the study included Swedish women aged 20 years or older at the year of diagnosis of breast cancer (International classification of diseases, seventh revision (ICD-7) code 170 as recorded in the National Cancer Register) between Jan. 1, 2015 and Dec. 31, 2019 and alive on Sep. 30, 2021. Women with breast cancer were divided into two groups based on their age at diagnosis of cancer (< 51, ≥ 51)^26,27^ to approximate premenopausal- and postmenopausal breast cancer groups. Patients in each BC subgroup were divided by PCR test information from Jan. 1, 2020, to Sep. 30, 2021 as follow: patients with a positive COVID-19 PCR test were categorized as having a COVID-19 diagnosis and those without a positive PCR test as not having a COVID-19 diagnosis (See Fig. 1). Sociodemographic data were extracted from the Longitudinal Integrated database for Health Insurance and Labour Market Studies at Statistics Sweden (LISA)^28^ and COVID-19 positive PCR tests data obtained from the National notifiable disease network (SmiNet)^29^. Data on history of drug use was extracted from the Swedish National Prescribed Drug Register^30^ in the period between Jan. 1, 2018 and Dec. 31, 2019. (See Fig. 1) Anatomical, Therapeutic, Chemical (ATC) codes were used to identify dispensed drugs. Although we searched for all ATC codes as concomitant prescriptions we just reported the ten most frequent dispensed drugs with their ATC codes in Supplementary Table 3. Data on specialist outpatient visits and admissions to hospital in the period between Jan. 1, 2015 and Dec. 31, 2019 (See Fig. 1) were obtained from the Swedish National Patient Register^31^. We used the International Classification of Diseases, tenth revision (ICD-10) codes to select the diagnosis of diseases for patients.

Age at breast cancer diagnosis (≥ 20 years, subcategorized by 10 years), country of birth (Sweden, Nordic countries except Sweden, 28 European Countries (EU28) except the Nordics, and outside EU28), marital status (married, not-married), educational level (primary (9 years), upper secondary (12 years), tertiary (> 12 years)), employment status (employed, unemployed/retired), year of breast cancer diagnosis (2015, 2016, 2017, 2018, 2019), history of drug use related to breast cancer (tamoxifen, letrozole, anastrozole, exemestanes, no use), were considered in association with COVID-19 diagnosis in the study. Data on marital status, educational level and employment status include the latest available data before the pandemic, generally in 2019. The number of outpatient hospital visits and the number of hospital admissions during five years prior to Jan. 1, 2020, were used as a proxy of patients’ health condition. The Charlson Comorbidity Index (CCI) was used as a summary index for comorbidity related to risk of mortality, classified as 0 (No comorbidity), 1 (CCI 1–2, Mild), 2 (CCI3-4, Moderate), and 3 (CCI ≥ 5, Severe). We determined the number of comorbidities from the Swedish National Patient Register, including data for both specialist outpatient visits and admission to hospital, for the period between 2015.01.01 and 2019.12.31. Therefore, the CCI used is an index of healthcare-related comorbidity burden based on the last prior 5 years before the pandemic and study index date (“5-year CCI”) and thus most (approximately 90%) but not all breast cancer patients will have 2 (malignancy) or 6 (metastatic malignancy) points of this “5-year CCI” if they have healthcare contacts in the last 5 years for their breast cancer or other cancers. To measure history of drug utilization, the number of different drugs dispensed during the period of Jan. 1, 2018 to Dec. 31, 2019 was calculated. History of drugs utilization was used as another proxy of patients’ health condition. We checked among the drugs dispensed in the two years before Jan. 1, 2020, to report the 10 most frequent dispensed drugs. We also assessed the frequency of some selected diseases in association with diagnosis of COVID-19: cardiovascular diseases, diabetes, and musculoskeletal system diseases based on appropriate ICD-10 codes (Supplementary Table 4) from specialist outpatient visits and admission to hospital in the Swedish National Patient Register during the period of data collection (2015.01.01-2019.12.31).

### Statistical analysis

Descriptive statistics including median and interquartile range (IQR), number and percentage of categorical variables were used to present sociodemographic characteristics, history of health service use and history of drug utilization of women with breast cancer between 2015 and 2019 and based on a positive PCR test of patients (called COVID-19 diagnosis/infection from now on). We report the results by menopausal status separately. T-tests for continuous variables and Chi-square tests for categorical variables were used to explore differences between patients with and without a COVID-19 diagnosis. We also calculated the difference between proportions with 95% confidence intervals to better explore the magnitude of such differences between patients with and without a COVID-19 diagnosis. To estimate the association between characteristics of patients and health condition with COVID-19 infection, logistic regression analyses were performed. Unadjusted and adjusted odds ratios (OR) with 95% confidence intervals (95%CI) were estimated. The adjustments included age at breast cancer diagnosis, marital status, educational level, and employment status. All analyses were performed using the SAS software, version 9.4.

## Results

We included 38,523 women with a breast cancer diagnosis during 5 years preceding the pandemic. The median age at diagnosis of breast cancer was 45 years (IQR = 40–48) for women with premenopausal breast cancer and 67 years (IQR = 60–73) for the postmenopausal group (see Supplementary Tables 1 and 2 for further characteristics of the women with breast cancer overall and by menopausal status, irrespective of COVID-19 diagnosis). Among women with premenopausal breast cancer, the prevalence of being unemployed/retired was 7% for women with a diagnosis of COVID-19 while it was 12.4% for women without a COVID-19 diagnosis (Table 1). Among women with a postmenopausal breast cancer, the proportion of unemployed/retired was 43.3% and 61.6% in women with versus without a COVID-19 diagnosis, respectively. While the proportions of women with premenopausal breast cancer who were born outside of EU28 were roughly the same for those with or without a COVID-19 infection (19.6% vs. 17.3% respectively), the proportions among postmenopausal women were 10.5% and 5.4% for women with versus without a COVID-19 diagnosis, respectively. We did not find any other significant difference in the characteristics of patients with or without a COVID-19 diagnosis neither among premenopausal women nor in postmenopausal group. The distribution of history of health care use (2015–2019) and drug utilization (2018–2019) among patients did not show a clear difference between women with or without a diagnosis of the COVID-19 in both premenopausal and postmenopausal women (Table 1). Characteristics of breast cancer patients, by menopausal status in Sweden are shown in supplementary Table 1. Drug and health service use among breast cancer patients by menopausal status is reported in supplementary Table 2. Regarding the prevalence of selected diseases, the study showed that women with a COVID-19 positive test have a statistically significant higher prevalence of out-patient visits due to ischemic heart failure or angina pectoralis among premenopausal breast cancer group. Among postmenopausal women, admission due to congestive heart failure, essential hypertension and COPD/Asthma were more prevalent among women with a COVID-19 diagnosis compared to women without a PCR positive test (Table 2). We used the Charlson Comorbidity Index (approximate quartiles) for comorbidities (2015–2019) as a surrogate for health status. Among women with postmenopausal BC, 12% of women in the test-positive COVID-19 group belong to the CCI medium and 24% to the severe category (Table 2), while the corresponding numbers were around 4% and 28%, respectively, among test-positive women with premenopausal BC (Table 2).

**Table 1:** Characteristics, history of health care use and drug utilization of breast cancer patients (2015–2019) comparing patients with a COVID-19 diagnosis and without a diagnosis by menopausal status in Sweden.

	Premenopausal (*N* = 7 892, 20.1%)				Postmenopausal (*N* = 30 631, 79.9%)			
	With a COVID-19 diagnosis (No = 2 588, 32.79%)	Without a COVID-19 diagnosis (No = 5 304, 67.21%)			With a COVID-19 diagnosis (No = 3 626, 11.82%)	Without a COVID-19 diagnosis (No = 27 005, 88.18%)		
	No. (%)	No. (%)	Difference (%)	95% confidence interval	No. (%)	No. (%)	Difference (%)	95% confidence interval
Age at breast cancer diagnosis								
20–30	87(3.4)	132(2.5)	0.9	0.1–1.7	---	---	---	---
31–40	654(25.3)	1132(21.3)	4.0	1.9–5.9	---	---	---	---
41–50	1847(71.4)	4040(76.2)	-4.8	(-6.9)- (-2.7)	---	---	---	---
51–60	---	---			1711(47.3)	6211(23.0)	24.2	22.5–25.2
61–70	---	---			916(25.3)	10,447(38.7)	-13.4	(-15.0)-(-11.9)
71–80	---	---			578(15.9)	7537(27.9)	-12.0	(-13.3)-(-10.7)
81–90	---	---			351(9.7)	2494(9.2)	0.4	(-0.6)-1.5
> 90	---	---			70(1.9)	316 (1.2)	0.8	0.3–1.2
Median of age (IQR)	44 (40–47)	45 (41–48)			61 (55–72)	68 (61–73)		
Range	21–50	20–50			51–99	51–101		
Country of birth								
Sweden	1 946(75.2)	4 081(76.9)	-1.7	(-3.8)-0.3	2 972(82.0)	23 317(86.4)	-4.4	(-5.7)-(-3.1)
Nordics excluding Sweden	41(1.6)	95(1.8)	-0.2	(-0.8)-0.4	167(4.6)	1 382(5.1)	-0.5	(-1.2)-0.2
Eu28 except the Nordics	94(3.6)	209(3.9)	-0.3	(-1.2)-0.6	106(2.9)	835(3.1)	-0.2	(-0.8)-0.4
Out of Eu28	507(19.6)	919(17.3)	2.3	0.4–4.1	381(10.5)	1 469(5.4)	5.1	4.0-6.1
Missing						2		
Marital status								
Married	1 474(57.0)	2 745(51.8)	5.2	2.9–7.5	1 862(51.4)	13 223(49.0)	2.4	0.7–4.1
Not married	1 114(43.0)	2 559(48.3)	-5.2	(-7.5)-(-2.9)	1 763(48.6)	13 780(51.0)	-2.4	(-4.1)-(-0.7)
Education level								
Primary (9 years)	152(5.9)	400(7.6)	-1.7	(-2.8)-(-0.5)	665(18.5)	5 877(21.9)	-3.4	(-4.8)-(-2.1)
Upper secondary (12 years)	972(37.7)	1 971(37.5)	0.4	(-1.9)-2.7	1 566(43.7)	11 630(43.4)	0.1	(-1.6)-1.8
Tertiary (More than 12 years)	1 454(56.4)	2 886(54.9)	1.8	(-0.6)-4.1	1 355(37.8)	9 312(34.7)	2.9	1.2–4.6
Missing	10	47			40	186		
Employment status								
Employed	2 407(93.0)	4 649(87.7)	5.4	4.0-6.7	2 054(56.7)	10 381(38.4)	18.2	16.5–19.9
Unemployed/retired	181(7.0)	655(12.4)	-5.4	(-6.7)-(-4.0)	1 571(43.3)	16 622(61.6)	-18.2	(-19.9)-(-16.5)
Diagnosis year								
2015	508(19.6)	1 060(20.0)	-0.4	(-2.2)-1.5	642(17.7)	5 008 (18.5)	-0.8	(-2.2)-0.5
2016	486(18.8)	1 059(20.0)	-1.2	(-3.0)-0.7	640(17.7)	5 080(18.8)	-1.2	(-2.5)-0.2
2017	505(19.5)	1 027(19.4)	0.2	(-1.7)-2.0	696(19.2)	5 432(20.1)	-0.9	(-2.3)-0.4
2018	486(18.8)	1 044(19.7)	-0.9	(-2.8)-0.9	757(20.9)	5 553(20.6)	0.3	(-1.1)-1.7
2019	603(23.3)	1 114(21.0)	2.3	0.3–4.3	891(24.6)	5 932(22.0)	2.6	1.1–4.1
History of Breast cancer related drug use*								
Tamoxifen	1 298(47.8)	2 617(46.7)	0.8	(-1.5)-3.2	939(23.5)	6 070(20.3)	3.4	1.9–4.9
Letrozole	177(6.5)	396(7.1)	-0.6	(-1.8)-0.6	1 012(25.3)	8 338(27.9)	-3.0	(-4.5)-(-1.4)
Anastrozole	86(3.2)	204(3.6)	-0.5	(-1.4)-0.3	573(14.4)	4 628(15.5)	-1.3	(-2.6)-(-0.1)
Exemestanes	39(1.4)	100(1.8)	-0.4	(-1.0)-0.2	151(3.8)	1 241(4.2)	-0.4	(-1.1)-0.3
No use	1 115(41.1)	2 292(40.9)	-0.1	(-2.5)-2.3	1 319(33.0)	9586(32.1)	0.9	(-0.8)-2.5
History of specialist outpatient visit (Any causes) *								
No visit	270(10.4)	610(11.5)	-1.1	(-2.5)-0.4	309 (8.5)	2468(9.1)	-0.6	(-1.6)-0.4
1–5	1 178(45.5)	2 495(47.0)	-1.5	(-3.9)-0.8	1 418(39.1)	11 203(41.5)	-2.4	(-4.1)-(-0.7)
6–9	562(21.7)	1 088(20.5)	1.2	(-0.7)-3.1	856(23.6)	6 179(22.9)	0.7	(-0.7)-2.2
10–15	235(9.1)	470(8.9)	0.2	(-1.1)-1.6	405(11.2)	3 210(11.9)	-0.7	(-1.8)-0.4
> 15	343(13.3)	641(12.1)	1.2	(-0.4)-2.7	638(17.6)	3 945(14.6)	3.0	1.7–3.4
History of hospitalization admissions (Any diagnosis) *								
Not admitted	1 621(62.6)	3461(65.3)	-2.6	(-4.9) -(-0.4)	2 044(56.4)	15 657(58.0)	-1.6	(-3.3)-0.1
1–5	938(36.2)	1799(33.9)	2.3	0.1–4.6	1 432(39.5)	10 680(39.6)	-0.1	(-1.8)-1.6
6–9	23(0.9)	38(0.7)	0.2	(-0.3)-0.6	111(3.1)	553(2.1)	1.0	0.4–1.6
10–15	4(0.2)	3(0.1)	0.1	(-0.1)-0.3	23(0.6)	90(0.3)	0.3	0.0-0.6
> 15	2(0.1)	3(0.1)	0.0	(-0.1)-0.1	16(0.4)	25(0.1)	0.3	0.1–0.6
History of dispensed drugs type**								
0	146(5.6)	411(7.8)	-2.1	(-3.3) -(-0.1)	102(2.8)	778(2.9)	-0.1	(-0.6)-0.5
1–4	822(31.8)	1 691(31.9)	-0.1	(-2.3)-2.1	730(20.1)	5 633(20.9)	-0.7	(-2.1)-0.7
5–9	768(29.7)	1 508(28.4)	1.2	(-0.9)-3.4	1 040(28.7)	8 440(31.3)	-2.6	(-4.1)-(-1.0)
10–15	450(17.4)	895(16.9)	0.5	(-1.3)-2.3	823(22.7)	6 124(22.7)	0.0	(-1.4)-1.5
> 15	402(15.5)	799(15.1)	0.5	(-1.2)-2.2	931(25.7)	6 030(22.3)	3.3	1.8–4.9
Age at the pandemic (1 Jan 2020)								
20–30	48(1.9)	74(1.4)	0.5	(-0.1)-1.1	---		---	---
31–40	421(16.3)	692(13.1)	3.2	1.5–4.9	---		---	---
41–50	1614(62.4)	3196(60.3)	2.1	(-0.2)-4.4	---		---	---
51–60	505(19.5)	1342(5.3)	-5.8	(-7.7) -(-3.9)	1374(37.9)	4386(16.2)	21.7	20,0–23,3
61–70	---	---		---	1039(28.7)	9191(34.0)	-5.4	(-7.0)-(-3.8)
71–80	---	---		---	691(19.1)	9584(35.5)	-16.4	(-17.8)-(-15.0)
81–90	---	---		---	386(10.7)	3236(12.0)	-1.3	(-2.4)-(-0.3)
> 90	---	---		---	136(3.8)	608(2.3)	1.5	0.9–2.1
Median of age (IQR)	46 (42–50)	48 (43–51)			64 (58–74)	70 (64–76)		
Average of age	24–55	20–55			51–100	51–104		

IQR: Interquartile range.

*History of health care use includes five years before the pandemic (1 Jan 2015-31 Dec 2019).

**History of drug utilization includes two years before the pandemic (1 Jan 2018-31 Dec 2019).

**Table 2:** History of selected diseases pre-baseline (2015–2019) in breast cancer patients comparing patients with and without COVID-19 diagnosis by menopausal status.

	Premenopausal				Postmenopausal			
	With a COVID-19 diagnosis (No = 2 588, 32.8%)	Without a COVID-19 diagnosis (No = 5 304, 67.2%)			With a COVID-19 diagnosis (No = 3 626, 11.8%)	Without a COVID-19 diagnosis (No = 27 005, 88.2%)		
	No. (%)	No. (%)	Difference (%)	95% confidence interval	No. (%)	No. (%)	Difference (%)	95% confidence interval
History of disease diagnosis in specialist outpatient visits								
Cardiovascular diseases	235 (9.1)	445(8.4)	0.7	(-0.6)-2.0	681(18.8)	5 133(19.0)	-0.2	(-1.6)-1.1
Diseases of veins. lymphatic vessels and lymph nodes. not elsewhere classified	132(5.1)	229(4.3)	0.8	(-0.2)-1.8	166(4.6)	1 190(4.4)	0.2	(-0.6)-0.9
Congestive heart failure	29(1.1)	56(1.1)	0.1	(-0.4)-0.6	144(4.0)	1 064(4.0)	0.0	(-0.6)-0.7
Atrial fibrillation	8(0.3)	11(0.2)	0.1	(-0.1)-0.3	129(3.6)	1 113(4.1)	-0.6	(-1.2)-0.1
Essential hypertension	19(0.8)	38(0.7)	0.0	(-0.4)-0.4	115(3.2)	870(3.2)	-0.1	(-0.7)-0.6
Stroke/transient ischemic attack	7(0.3)	12(0.3)	0.0	(-0.2)-0.3	67(1.9)	458(1.7)	0.2	(-0.3)-0.6
Ischemic heart diseases	9(0.4)	6(0.1)	0.2	0.0-0.5	88(2.5)	624(2.3)	0.1	(-0.4)-0.6
Cerebrovascular diseases	4(0.2)	14(0.3)	-0.1	(-0.3)-0.1	50(1.4)	357(1.3)	0.1	(-0.3)-0.5
Diseases of arteries. arterioles and capillaries	19(0.8)	42(0.8)	-0.1	(-0.5)-0.3	71(2.0)	442(1.7)	0.3	(-0.2)-0.8
Peripheral arterial disease	7 (0.3)	22(0.4)	-0.1	(-0.4)-0.1	56(1.6)	336(1.3)	0.3	(-0.1)-0.7
Pulmonary heart disease and diseases of pulmonary circulation	10(0.4)	33(0.6)	-0.2	(-0.6)-0.1	35(1.0)	313(1.2)	-0.2	(-0.5)-0.1
Myocardial infarction	1(0.1)	3(0.1)	0.0	(-0.1)-0.1	40(1.1)	238(0.9)	0.2	(-0.1)-0.6
Angina pectoris	6(0.3)	3(0.1)	0.2	0.0-0.4	38(1.1)	285(1.1)	0.0	(-0.4)-0.3
Diabetes								
Diabetes type 2	8(0.3)	22(0.4)	-0.1	(-0.4)-0.2	71(2.0)	496(1.9)	0.1	(-0.4)-0.6
Respiratory system								
COPD/Asthma	37(1.5)	56(1.1)	0.4	(-0.2)-0.9	99(2.8)	599(2.2)	0.5	0.0-1.1
Musculoskeletal system								
Arthrosis	56(2.2)	100(1.9)	0.3	(-0.4)-0.9	391(10.8)	2 826(10.5)	0.3	(-0.8)-1.4
Inflammatory polyarthropathies	24(1.0)	52(1.0)	-0.1	(-0.5)-0.4	81(2.3)	578(2.2)	0.1	(-0.4)-0.6
Systemic connective tissue disorders	12(0.5)	32(0.6)	-0.1	(-0.5)-0.2	35(1.0)	290(1.1)	-0.1	(-0.4)-0.2
Infectious arthropathies	2(0.1)	6(0.1)	0.0	(-0.2)-0.1	11(0.3)	58(0.2)	0.1	(-0.1)-0.3
History of disease diagnosis in admitted patients								
Cardiovascular diseases	40(1.6)	70(1.3)	0.2	(-0.3)-0.8	326(9.0)	2 445(9.1)	-0.1	(-1.1)-0.9
Diseases of veins. lymphatic vessels and lymph nodes, not Elsewhere classified	4(0.2)	17(0.3)	-0.2	(-0.4)-0.0	17(0.5)	117(0.5)	0.0	(-0.2)-0.3
Congestive heart failure	6(0.3)	13(0.3)	0.0	(-0.2)-0.2	90(2.5)	481(1.8)	0.7	0.2–1.2
Atrial fibrillation	4(0.2)	3(0.1)	0.1	(-0.1)-0.3	68(1.9)	556(2.1)	-0.2	(-0.7)-0.3
Essential hypertension	1 (0.1)	2(0.1)	0.0	(-0.1)-0.1	19(0.5)	84(0.3)	0.2	0.0-0.5
Stroke/transient ischemic attack	8(0.3)	8(0.2)	0.2	(-0.1)-0.4	94(2.6)	665(2.5)	0.1	(-0.4)-0.7
Ischemic heart diseases	6(0.3)	6(0.1)	0.1	(-0.1)-0.3	62(1.7)	457(1.7)	0.0	(-0.4)-0.5
Cerebrovascular diseases	6(0.3)	6(0.1)	0.1	(-0.1)-0.3	73(2.0)	487(1.8)	0.2	(-0.3)-0.7
Diseases of arteries. arterioles and capillaries	3(0.1)	3(0.1)	0.1	(-0.1)-0.2	19(0.5)	146(0.6)	0.0	(-0.3)-0.2
Peripheral arterial disease	1(0.1)	4(0.1)	0.0	(-0.1)-0.1	17(0.5)	144(0.6)	-0.1	(-0.3)-0.2
Pulmonary heart disease and diseases of pulmonary circulation	4(0.2)	20(0.4)	-0.2	(-0.4)-0.0	41(1.2)	331(1.3)	-0.1	(-0.5)-0.3
Myocardial infarction	2(0.1)	6(0.1)	0.0	(-0.2)-0.1	45(1.3)	312(1.2)	0.1	(-0.3)-0.5
Angina pectoris	3(0.1)	3(0.1)	0.1	(-0.1)-0.2	17(0.5)	140(0.5)	0.0	(-0.3)-0.2
Diabetes								
Diabetes type 2	3(0.1)	6(0.1)	0.0	(-0.2)-0.2	14(0.4)	59(0.2)	0.2	0.0-0.4
Respiratory system								
COPD/Asthma	2(0.1)	1(0.0)	0.1	(-0.1)-0.2	49(1.4)	225(0.9)	0.5	0.1–0.9
Musculoskeletal system								
Arthrosis	10(0.4)	29(0.6)	-0.2	(-0.5)-0.2	145(4.0)	1199(4.5)	-0.4	(-1.1)-0.2
Inflammatory polyarthropathies	2(0.1)	3(0.1)	0.0	(-0.1)-0.1	15(0.4)	65(0.3)	0.2	0.0-0.4
Systemic connective tissue disorders	3(0.1)	2(0.1)	0.1	(-0.1)-0.2	5(0.2)	56(0.2)	-0.1	(-0.2)-0.1
Infectious arthropathies	2(0.1)	2(0.1)	0.0	(-0.1)-0.2	5(0.2)	40(0.2)	0.0	(-0.1)-0.1
Charlson Comorbidity Index								
0 (no comorbidity)	246 (9.5)	494 (9.3)	0.2	(-1.2)-1.6	219 (6.0)	1430 (5.3)	0.7	(-0.1)-1.6
1–2 (Mild)	1499 (57.9)	3042 (57.4)	0.6	(-1.8)-2.9	2117 (58.3)	16,746 (62.0)	-3.6	(-5.3.)-1.9
3–4 (Moderate)	107 (4.1)	189 (3.6)	0.6	(-0.3)-1.5	428 (11.8)	2815 (10.4)	1.4	0.3–2.5
≥ 5 (Severe)	736 (28.4)	1579 (29.8)	-1.3	(-3.5)-0.8	862 (23.8)	6014 (22.3)	1.5	(0.0–3.0

Unadjusted and adjusted odd ratios (OR) of factors associated with diagnosis of COVID-19 and their 95% Confidence Intervals (95% CI) are presented in Table 3. Among premenopausal women, COVID-19 diagnosis was associated with being born outside of EU28 OR = 1.29 (95%CI: 1.13–1.46), being married OR = 1.23 (95%CI: 1.12–1.36), having upper secondary school education OR = 1.25 (95%CI: 1.01–1.54) and unemployment status OR = 1.92 (95%CI: 1.59–2.30). Patients with more than 15 outpatient visits or admitted to hospital 1–5 times in the 5 years prior to Jan. 1, 2020 (Table 3, Supplementary Fig. 1) had higher odds of COVID-19 diagnosis (OR = 1.31 (95%CI:1.07–1.61) and OR = 1.12 (95%CI:1.01–1.25) respectively). Adjusted odds ratios of diagnosis of COVID-19 increased by increasing the number of dispensed drugs from OR = 1.36 (95%CI:1.10–1.67) among patients with 1–5 drugs to OR = 1.53 (95%CI: 1.21–1.93) in patients with > 15 drugs (Table 3, Supplementary Fig. 1).

**Table 3:** Unadjusted and adjusted odds ratio and 95% confidence interval of diagnosis of COVID-19 for a selection of patient characteristics, by menopausal breast cancer status.

			Unadjusted^*^		Adjusted^**^	
	Cov+	Cov-	OR	95% CI	OR	95% CI
Premenopausal breast cancer						
Palace of birth						
Sweden	1 946	4 081	Ref	Ref	Ref	Ref
Nordics excluding Sweden	41	95	0.91	0.63–1.31	0.99	0.68–1.44
Eu28 except the Nordics	94	209	0.94	0.74–1.21	1.01	0.78–1.30
Out of Eu28	507	919	1.16	1.03–1.31	1.29	1.13–1.46
Marital status						
Married	1 474	2 745	1.23	1.12–1.36	1.23	1.12–1.36
Not married	1 114	2 559	Ref	Ref	Ref	Ref
Education level						
Primary (9 years)	152	400	Ref	Ref	Ref	Ref
Upper secondary (12 years)	972	1 971	1.30	1.06–1.59	1.25	1.01–1.54
Tertiary (More than 12 years)	1 454	2 886	1.33	1.09–1.62	1.20	0.98–1.48
Employment status						
Employed	2 407	4 649	Ref	Ref	Ref	Ref
Unemployed	181	655	1.87	1.58–2.23	1.92	1.59–2.30
No. of outpatient visits (any cause)						
Not visited specialist clinics	270	610	Ref	Ref	Ref	Ref
1–5	1 178	2 495	1.07	0.91–1.25	1.04	0.89–1.23
6–9	562	1 088	1.17	0.98–1.39	1.18	0.99–1.41
10–15	235	470	1.13	0.91–1.40	1.16	0.93–1.44
> 15	343	641	1.21	1.00-1.47	1.31	1.07–1.61
No. of admissions (any diagnosis)						
Not admitted	1 621	3 461	Ref	Ref	Ref	Ref
1–5	938	1 799	1.11	1.01–1.23	1.12	1.01–1.25
6–9	23	38	1.29	0.77–2.18	1.45	0.85–2.48
10–15	4	3	2.84	0.64–12.72	3.79	0.81–17.65
> 15	2	3	1.42	0.24–8.53	3.40	0.47–24.79
No. of dispensed drugs type						
0	146	411	Ref	Ref	Ref	Ref
1–5	822	1 691	1.37	1.11–1.68	1.36	1.10–1.67
6–9	768	1 508	1.43	1.17–1.77	1.44	1.17–1.78
10–15	450	895	1.42	1.14–1.76	1.46	1.17–1.83
> 15	402	799	1.42	1.13–1.77	1.53	1.21–1.93
Postmenopausal breast cancer						
Palace of birth						
Sweden	2 972	23 317	Ref	Ref	Ref	Ref
Nordics excluding Sweden	167	1 382	0.95	0.80–1.12	1.03	0.87–1.22
Eu28 except the Nordics	106	835	1.00	0.81–1.22	0.94	0.76–1.16
Out of Eu28	381	1 469	2.04	1.81–2.29	1.61	1.41–1.83
Marital status						
Married	1 862	13 223	1.10	1.03–1.18	1.12	1.04–1.21
Not married	1 763	13 780	Ref	Ref	Ref	Ref
Education level						
Primary (9 years)	665	5 877	Ref	Ref	Ref	Ref
Upper secondary (12 years)	1 566	11 630	1.19	1.08–1.31	1.02	0.92–1.13
Tertiary (More than 12 years)	1 355	9 312	1.29	1.17–1.42	1.01	0.90–1.12
Employment status						
Employed	2 054	10 381	Ref	Ref	Ref	Ref
Unemployed	1 571	16 622	2.09	1.95–2.46	1.54	1.40–1.69
No. of outpatient visits (Any cause)						
Not visited specialist clinics	309	2 468	Ref	Ref	Ref	Ref
1–5	1 418	11 203	1.01	0.89–1.15	1.03	0.90–1.18
6–9	856	6 179	1.11	0.96–1.27	1.22	1.06–1.41
10–15	405	3 210	1.01	0.86–1.18	1.14	0.97–1.35
> 15	638	3 945	1.29	1.12–1.49	1.47	1.26–1.71
No. of admissions (Any diagnosis)						
Not admitted	2 044	15 657	Ref	Ref	Ref	Ref
1–5	1 432	10 680	1.03	0.96–1.10	1.15	1.06–1.24
6–9	111	553	1.54	1.25–1.89	1.94	1.55–2.42
10–15	23	90	1.96	1.24–3.10	2.48	1.55–3.98
> 15	16	25	4.91	2.61–9.20	6.35	3.33–12.11
No. of dispensed drugs type						
0	102	778	Ref	Ref	Ref	Ref
1–5	730	5 633	0.99	0.79–1.23	1.09	0.86–1.36
6–9	1 040	8 440	0.94	0.76–1.17	1.15	0.92–1.43
10–15	823	6 124	1.03	0.82–1.28	1.33	1.06–1.66
> 15	931	6 030	1.18	0.95–1.47	1.58	1.26–1.99

*Each characteristic was analyzed in a separate model.

*Adjusted for age at breast cancer diagnosis. marital status. educational level, employment status and charlson comorbidity index where applicable.

Among postmenopausal women, being diagnosed with COVID-19 was associated with being born outside of EU28 OR = 1.61 (95%CI: 1.41–1.83), being married OR = 1.12 (95%CI: 1.04–1.21), and being unemployed OR = 1.54 (95%CI: 1.40–1.69) (Table 3). Moreover, diagnosis of COVID-19 was associated with increasing number of outpatient visits as well as number of hospital admissions (Table 3, Supplementary Fig. 1): the OR of having a COVID-19 diagnosis increased from OR = 1.22 (95%CI: 1.06–1.41) for 6–9 outpatient visits to OR = 1.47 (95%CI: 1.26–1.71) for > 15 and from OR = 1.15 (95%CI: 1.06–1.24) for patients with 1–5 hospital admission to OR = 6.35 (95%CI: 3.33–12.11) for patients with > 15 hospital admission. An elevated odd of diagnosis of COVID-19 associated with a higher number of drugs dispensed prior to the pandemic was seen: from OR = 1.09 (95%CI: 0.86–1.36) for patients with 1–5 drugs to OR = 1.58 (95%CI: 1.26–1.99) for patients who had used > 15 drugs. The ten most frequently dispensed drugs two years prior to the pandemic (2018–2019) among breast cancer patients for both pre- and postmenopausal women, comparing those with a diagnosis of COVID-19 and those without the diagnosis, are reported in Supplementary Table 3. Among postmenopausal women, 56.3% of patients with a diagnosis of COVID-19 had analgesics dispensed, while the proportion was 50.9% among patients without the diagnosis in the same menopausal status. No other drug classes showed significant differences for patients subsequently infected with COVID-19.

## Discussion

The results of our study showed women with premenopausal- or postmenopausal breast cancer who developed COVID-19 disease during the study period had higher probability of being born outside of EU28, being married, or being unemployed compared to women without the COVID-19. Moreover, women with postmenopausal breast cancer who developed COVID-19 were more likely to have outpatient visits or hospital admissions in the years preceding the pandemic. Furthermore, women in both menopausal groups of breast cancer with COVID-19 were more likely to have higher drug utilization in their history compared to women without COVID-19.

Our finding that foreign-born women with breast cancer were infected to a larger extent aligns with studies from general populations reporting that the risk of COVID-19 is higher among immigrants than native people in many places^32–34^. It is likely that cultural background of born place may influence the health service care use which in turn leads to a poorer general health condition for the women. However, it is important to recognize that people who were born outside of EU28 are not a homogenous community and the term covers very different geographical areas: from the US, Canada, and Australia, which are relatively similar to Sweden in culture and interpersonal relation patterns, to sub-Saharan countries or the middle east countries with less similarity in culture and interpersonal relation patterns compared to Sweden. Guijarro et al. has shown that immigrants from sub-Saharan countries are at higher risk of COVID-19 compared to people who were born in Europe or who immigrated to European countries from North African countries or Asia^32^. The heterogeneity of the immigrant population is also important to keep in mind since breast cancer is a disease with socioeconomic gradients and has its highest frequency in the developed industrial countries^35^.

Our findings that unemployed status is a risk factor for COVID-19 diagnosis are not consistent with some other studies, possibly because of the way that we assessed employment status. In our study, we combined the unemployed and retired people into the same group. In this case, the results from the premenopausal group would not change because all are younger than 51. However, many of the women in the postmenopausal group would be retired because this group contains women who are ≥ 51 years of age. Since we did not have any variable to separate between the two groups and an age cut-off would have resulted in almost the same subcategories we were not able to perform any extra analysis. Moreover, in this study, the employed category is a composite relative to unemployed whereas many other studies report their results for employed persons based on subcategories of employment status: e.g. essential occupations, defined as “*critical occupations that are necessary for the functioning of societal infrastructures*,* demand on-site labor and involves close proximity with members of the public and coworkers*”^36^ and non-essential occupations (other occupations than essential occupations). For instance, Nwaru et al.^37^ reported, based on the same data source as this study, that working in essential occupations was related to higher risk of COVID-19 diagnosis, especially for workers in the healthcare sectors. Similar results were reported by Mutambudzi et al.^38^, where health workers had a more than seven-fold greater risk of severe COVID-19 while other essential workers had a slightly higher risk compared to non-essential workers.

Higher consultation rates and higher levels of dispensed drugs prior to the pandemic among women diagnosed with COVID-19 are not surprising. These measures are reasonable proxies for the patients’ multimorbidity and health status. A previous British study showed that number of prescribed drugs is the most powerful predictor of future health consultations and the second most powerful predictor of mortality^39^ .We did not find any study that investigated the association of history of drug use associated with of COVID-19 diagnosis in breast cancer patients. In addition, it is important to consider that multimorbidity and polypharmacy are more frequent among elderly people^40^, which is consistent with the breast cancer population and our study population (75% of population in our study were ≥ 65-years old).

Our study has several strengths. We have access to a comprehensive database on COVID-19-tests, hospital diagnoses, dispensed prescription drugs and sociodemographic data for all patients in Sweden. Moreover, data on all Swedish women diagnosed with breast cancer was available with full national level coverage from the Swedish National Cancer Register. Our study had some limitations as well. First, second generation immigrants who were born in Sweden were considered as people born in Sweden, which might dilute the results when we use place of birth as a proxy for cultural and/or genetic ethnicity differences. Second, we used number of consultations, health service use and drug consumption as a proxy to show patients’ health condition due to their physical-, psychological- or social unmet needs because there is no direct data on patient frailty. There may also be some misclassification of diagnoses and drugs, particularly under ascertainment, since data on diagnoses in primary care and drugs administered in hospitals were not available in this analysis. Third, the CCI used in the manuscript is based on the diagnoses for each individual within the recent 5 years (2015–2019). Some women had CCI < 2 because their breast cancer was diagnosed earlier than the time period and thus did not contribute to their CCI. The 5-year CCI is still relevant because it reflects the concurrent health status of the women included in the study, and a recent breast cancer is part of that health burden. It is also more comparable to CCI calculated for other patients, since it uses the standard components. Last, since lacking a variable to show the menopausal status, we have used age at 50 as a cut-off to separate the premenopausal and postmenopausal breast cancers. Selecting the cut-off can induce a non-differential misclassification of the menopausal status, since menopause could happen between 44 and 55 ^27^, which would tend to dilute any differences between the pre- and post-menopausal groups. Further research is needed to cover the limitations mentioned.

## Conclusion

Our study demonstrates that some characteristics of women with premenopausal- or postmenopausal breast cancer such as unemployment, country of birth or health status measured by number of prescribed drugs were more prevalent among women who developed COVID-19 compared to women without COVID-19 diagnosis. It may emphasize the need to support patients / improve access to services for patients with an immigrant background. No major difference in other characteristics were seen between premenopausal- and postmenopausal breast cancer patients.

## Electronic supplementary material

Below is the link to the electronic supplementary material.

Supplementary File 1

## Data Availability

The data underlying this article cannot be shared publicly due to their containing sensitive information that could compromise the privacy of research participants. Analysis data will be shared upon reasonable request to the corresponding author (Mohammadhossein Hajiebrahimi). Access to similar data requires permission. Apart from ethical approval from the Swedish Ethical Review Authority, researchers will also need approval from each register holder.

## Abbreviations

ATC: Anatomical, therapeutic, chemical classification system
BC: Breast cancer
EU28: European Union consists a group of 28 countries
COVID-19: Coronavirus Disease 2019
ICD-7: International classification of diseases, seventh revision
ICD-10: International classification of diseases, tenth revision
LISA: Longitudinal Integrated database for Health Insurance and Labour Market
PCR: Polymerase chain reaction
SCIFI-PEARL: Swedish COVID-19 Investigation for future insights – a population epidemiology approach using register linkage
SmiNet: Sweden national notifiable disease network
