# Supplementary material for: Sociodemographic characteristics and health status of women with breast cancer and COVID 19 diagnosis by menopausal status a cross sectional study
Source: Sci Rep. 2025 Jan 21;15:2648. doi: 10.1038/s41598-025-86710-8 (PMC11751173; doi:10.1038/s41598-025-86710-8)
Supplement: Supplementary file 1 [file 41598_2025_86710_MOESM1_ESM.docx]

***Supplement to:***

**Sociodemographic characteristics and health status of women with breast cancer and COVID-19 diagnosis by menopausal status, A cross-sectional study**

**Mohammadhossein Hajiebrahimi^1*^, Hussam Shihan^2^, Ola Bratt^3^, Huiqi Li ^4^, Fredrik Nyberg^4^, Björn Wettermark^1^**

^1^ Department of Pharmacy, Faculty of Pharmacy, Uppsala University, Uppsala, Sweden

^2^ Clincal Studies Department, University Hospital, Linköping, Region Östergötland, Sweden

^3^ Department of Urology, Institute of Clinical Sciences, Sahlgrenska Academy, University of Gothenburg, Sweden

^4^ School of Public Health and Community Medicine, Institute of Medicine, Sahlgrenska Academy, University of Gothenburg, Gothenburg, Sweden.

| **Supplementary Table 1: Characteristics of breast cancer patients, by menopausal status in Sweden** | | | | | | |
| --- | --- | --- | --- | --- | --- | --- |
|  | **Total**  (No=38 523) | | **Premenopausal**  (N=7 892, 20.1%) | | **Postmenopausal**  (N=30 631, 79.9%) | |
| **Variables** | **No.** | **(%)** | **No.** | **(%)** | **No.** | **(%)** |
| **Age at breast cancer diagnosis** |  |  |  |  |  |  |
| 20-30 | 219 | 0.6 | 219 | 2.8 | --- | --- |
| 31-40 | 1786 | 4.6 | 1786 | 22.6 | --- | --- |
| 41-50 | 5887 | 15.3 | 5887 | 74.6 | --- | --- |
| 51-60 | 7922 | 20.6 | --- | --- | 7922 | 25.9 |
| 61-70 | 11363 | 29.5 | --- | --- | 11363 | 37.1 |
| 71-80 | 8115 | 21.1 | --- | --- | 8115 | 26.5 |
| 81-90 | 2845 | 7.4 | --- | --- | 2845 | 9.3 |
| >90 | 386 | 1.0 | --- | --- | 386 | 1.3 |
| Median (IQR) | 64 (53-72) |  | 45 (40-48) |  | 67 (60-73) |  |
| Range | 20-101 |  | 20-50 |  | 51-101 |  |
| **Country of birth** |  |  |  |  |  |  |
| Sweden | 32 316 | 83.9 | 6 027 | 76.4 | 26 289 | 85.8 |
| Nordics excluding Sweden | 1 685 | 4.4 | 136 | 1.7 | 1 549 | 5.1 |
| Eu28 except the Nordics | 1 244 | 3.2 | 303 | 3.8 | 941 | 3.1 |
| Out of Eu28 | 3 276 | 8.5 | 1 426 | 18.1 | 1 850 | 6.0 |
| Missing | 2 |  |  |  | 2 |  |
| **Marital status** |  |  |  |  |  |  |
| Married | 19 304 | 50.1 | 4 219 | 53.5 | 15 085 | 49.3 |
| Not married | 19 216 | 49.9 | 3 673 | 46.5 | 15 543 | 50.8 |
| Missing | 3 |  |  |  | 3 |  |
| **Education level** |  |  |  |  |  |  |
| Primary (9 years) | 7 094 | 18.6 | 552 | 7.1 | 6 542 | 21.5 |
| Upper secondary (12 years) | 16 139 | 42.2 | 2 943 | 37.6 | 13 196 | 43.4 |
| Tertiary (More than 12 years) | 15 007 | 39.2 | 4 340 | 55.4 | 10 667 | 35.1 |
| Missing | 283 |  | 57 |  | 226 |  |
| **Employment status** |  |  |  |  |  |  |
| Employed | 19 491 | 50.6 | 7 056 | 89.4 | 12 435 | 40.6 |
| Unemployed | 19 029 | 49.4 | 836 | 10.6 | 18 193 | 59.4 |
| Missing | 3 |  |  |  | 3 |  |
| **Diagnosis year** |  |  |  |  |  |  |
| 2015 | 7 218 | 18.7 | 1 568 | 19.9 | 5 650 | 18.5 |
| 2016 | 7 265 | 18.9 | 1 548 | 19.6 | 5 720 | 18.7 |
| 2017 | 7 660 | 19.9 | 1 532 | 19.4 | 6 128 | 20.0 |
| 2018 | 7 840 | 20.4 | 1 530 | 19.4 | 6 310 | 20.6 |
| 2019 | 8 540 | 22.2 | 1 717 | 21.8 | 6 823 | 22.3 |
| **History of Breast cancer related drug use*** | |  |  |  |  |  |
| Tamoxifen | 10 924 | 25.9 | 3 915 | 47.0 | 7 009 | 20.7 |
| Letrozole | 9 923 | 23.5 | 573 | 6.9 | 9 350 | 27.6 |
| Anastrozole | 5 491 | 13.0 | 290 | 3.5 | 5 201 | 15.4 |
| Exemestanes | 1 531 | 3.6 | 139 | 1.7 | 1 392 | 4.1 |
| No use | 14 312 | 33.9 | 3 407 | 40.9 | 10 905 | 32.2 |
| **Age at start of the pandemic (1 Jan 2020)** | |  |  |  |  |  |
| 20-30 | 122 | 0.3 | 122 | 1.6 | --- | --- |
| 31-40 | 1113 | 2.9 | 1113 | 14.1 | --- | --- |
| 41-50 | 4810 | 12.5 | 4810 | 61.0 | --- | --- |
| 51-60 | 7607 | 19.8 | 1847 | 23.4 | 5760 | 18.8 |
| 61-70 | 10230 | 26.6 | --- | --- | 10230 | 33.4 |
| 71-80 | 10275 | 26.7 | --- | --- | 10275 | 33.5 |
| 81-90 | 3622 | 9.4 | --- | --- | 3622 | 11.8 |
| >90 | 744 | 1.9 | --- | --- | 744 | 2.4 |
| Median (IQR) | 66 (55-74) |  | 47 (43-50) |  | 70 (63-76) |  |
| Range | 20-104 |  | 20-55 |  | 51-104 |  |
| IQR: Interquartile range  **History includes five years before the pandemic (1 Jan 2015-31 Dec 2019)* | | | | |  | |

| **Supplementary Table 2: History of health care use in the 5 years before baseline and drug utilization in the 2 years before baseline among breast cancer patients in Sweden 1 Jan 2020, by menopausal status** | | | | | | |
| --- | --- | --- | --- | --- | --- | --- |
|  | **Total**  (N=38 523) | | **Premenopausal**  (N=7 892, 20.1%) | | **Postmenopausal**  (N=30 631, 79.9%) | |
| **No. of outpatient visits** | **No.** | **(%)** | **No.** | **(%)** | **No.** | **(%)** |
| No visit | 3 657 | 9.5 | 880 | 11.2 | 2 777 | 9.1 |
| 1-5 | 16 294 | 42.3 | 3 673 | 46.5 | 12 621 | 41.2 |
| 6-9 | 8 685 | 22.5 | 1 650 | 20.9 | 7 035 | 23.0 |
| 10-15 | 4 320 | 11.2 | 705 | 8.9 | 3 615 | 11.8 |
| ≥15 | 5 567 | 14.5 | 984 | 12.5 | 4 583 | 14.9 |
| **No. of admissions (any diagnosis)** | | | |  |  |  |
| Not admitted | 22 783 | 59.1 | 5 082 | 64.4 | 17 701 | 57.8 |
| 1-5 | 14 849 | 38.6 | 2 737 | 34.7 | 12 112 | 39.5 |
| 6-9 | 725 | 1.9 | 61 | 0.8 | 664 | 2.2 |
| 10-15 | 120 | 0.3 | 7 | 0.1 | 113 | 0.4 |
| ≥16 | 46 | 0.1 | 5 | 0.1 | 41 | 0.2 |
| **No. of dispensed drugs type** | | | |  |  |  |
| 0 | 1 437 | 3.7 | 557 | 7.1 | 880 | 2.9 |
| 1-4 | 8 876 | 23.0 | 2 513 | 31.8 | 6 363 | 20.8 |
| 5-9 | 11 756 | 30.5 | 2 276 | 28.8 | 9 480 | 31.0 |
| 10-14 | 8 292 | 21.5 | 1 345 | 17.0 | 6 947 | 22.7 |
| ≥15 | 8 162 | 21.2 | 1 201 | 15.2 | 6 961 | 22.7 |

| **Supplementary Table 3: History of ten most frequent drugs dispensed pre-baseline (2018-2019) among breast cancer patients in Sweden 1 Jan 2020, by menopausal status and COVID-19** | | | | | | | |
| --- | --- | --- | --- | --- | --- | --- | --- |
| **Patients with a COVID-19 diagnosis** | | | | **Patients without a COVID-19 diangosis** | | | |
| **Medicine name** | **ATC** | **No.** | **%** | **Medicine name** | **ATC** | **No.** | **%** |
| **Premenopausal** |  |  |  |  |  |  |  |
| [Analgesics](https://www.whocc.no/atc_ddd_index/?code=N02A&showdescription=yes) | N02 | 1307 | 50.5 | [Analgesics](https://www.whocc.no/atc_ddd_index/?code=N02A&showdescription=yes) | N02 | 2614 | 49.3 |
| [Antibacterial for systemic use](https://www.whocc.no/atc_ddd_index/?code=J01&showdescription=no) | J01 | 1278 | 49.4 | [Antibacterial for systemic use](https://www.whocc.no/atc_ddd_index/?code=J01&showdescription=no) | J01 | 2437 | 46.0 |
| [Anti-inflammatory and antirheumatic products](https://www.whocc.no/atc_ddd_index/?code=M01&showdescription=no) | M01 | 842 | 32.5 | [Anti-inflammatory and antirheumatic products](https://www.whocc.no/atc_ddd_index/?code=M01&showdescription=no) | M01 | 1743 | 32.9 |
| [Psycholeptics](https://www.whocc.no/atc_ddd_index/?code=N05&showdescription=no) | N05 | 808 | 31.2 | [Psycholeptics](https://www.whocc.no/atc_ddd_index/?code=N05&showdescription=no) | N05 | 1665 | 31.4 |
| [Drugs for acid related disorders](https://www.whocc.no/atc_ddd_index/?code=A02&showdescription=no) | A02 | 787 | 30.4 | [Drugs for acid related disorders](https://www.whocc.no/atc_ddd_index/?code=A02&showdescription=no) | A02 | 1550 | 29.2 |
| [Corticosteroids for systemic use](https://www.whocc.no/atc_ddd_index/?code=H02&showdescription=no) | H02 | 706 | 27.3 | [Corticosteroids for systemic use](https://www.whocc.no/atc_ddd_index/?code=H02&showdescription=no) | H02 | 1379 | 26.0 |
| [Drugs for constipation](https://www.whocc.no/atc_ddd_index/?code=A06&showdescription=no) | A06 | 655 | 25.3 | [Psycho analeptics](https://www.whocc.no/atc_ddd_index/?code=N06&showdescription=no) | N06 | 1307 | 24.6 |
| [Corticosteroids. dermatological preparations](https://www.whocc.no/atc_ddd_index/?code=D07&showdescription=no) | D07 | 653 | 25.2 | [Corticosteroids, dermatological preparations](https://www.whocc.no/atc_ddd_index/?code=D07&showdescription=no) | D07 | 1248 | 23.5 |
| [Psycho analeptics](https://www.whocc.no/atc_ddd_index/?code=N06&showdescription=no) | N06 | 618 | 23.9 | [Drugs for constipation](https://www.whocc.no/atc_ddd_index/?code=A06&showdescription=no) | A06 | 1230 | 23.2 |
| [Antihistamines for systemic use](https://www.whocc.no/atc_ddd_index/?code=R06&showdescription=no) | R06 | 589 | 22.8 | [Antihistamines for systemic use](https://www.whocc.no/atc_ddd_index/?code=R06&showdescription=no) | R06 | 1121 | 21.1 |
| **Postmenopausal** |  |  |  |  |  |  |  |
| [Analgesics](https://www.whocc.no/atc_ddd_index/?code=N02A&showdescription=yes) | N02 | 2041 | 56.3 | [Analgesics](https://www.whocc.no/atc_ddd_index/?code=N02A&showdescription=yes) | N02 | 13752 | 50.9 |
| [Antibacterial for systemic use](https://www.whocc.no/atc_ddd_index/?code=J01&showdescription=no) | J01 | 1875 | 51.7 | [Antibacterial for systemic use](https://www.whocc.no/atc_ddd_index/?code=J01&showdescription=no) | J01 | 12613 | 46.7 |
| [Psycholeptics](https://www.whocc.no/atc_ddd_index/?code=N05&showdescription=no) | N05 | 1340 | 37.0 | [Agents acting on the renin-angiotensin system](https://www.whocc.no/atc_ddd_index/?code=C09&showdescription=no) | C09 | 10128 | 37.5 |
| [Drugs for acid related disorders](https://www.whocc.no/atc_ddd_index/?code=A02&showdescription=no) | A02 | 1330 | 36.7 | [Psycholeptics](https://www.whocc.no/atc_ddd_index/?code=N05&showdescription=no) | N05 | 9698 | 35.9 |
| [Agents acting on the renin-angiotensin system](https://www.whocc.no/atc_ddd_index/?code=C09&showdescription=no) | C09 | 1198 | 33.0 | [Drugs for acid related disorders](https://www.whocc.no/atc_ddd_index/?code=A02&showdescription=no) | A02 | 9275 | 34.4 |
| [Drugs for constipation](https://www.whocc.no/atc_ddd_index/?code=A06&showdescription=no) | A06 | 1115 | 30.8 | [Mineral supplements](https://www.whocc.no/atc_ddd_index/?code=A12&showdescription=no) | A12 | 8869 | 32.8 |
| [Antithrombotic agents](https://www.whocc.no/atc_ddd_index/?code=B01&showdescription=no) | B01 | 1111 | 30.6 | [Antithrombotic agents](https://www.whocc.no/atc_ddd_index/?code=B01&showdescription=no) | B01 | 8371 | 31.0 |
| [Anti-inflammatory and antirheumatic products](https://www.whocc.no/atc_ddd_index/?code=M01&showdescription=no) | M01 | 1111 | 30.6 | [Beta blocking agents](https://www.whocc.no/atc_ddd_index/?code=C07&showdescription=no) | C07 | 7692 | 28.5 |
| [Mineral supplements](https://www.whocc.no/atc_ddd_index/?code=A12&showdescription=no) | A12 | 1091 | 30.1 | [Corticosteroids. dermatological preparations](https://www.whocc.no/atc_ddd_index/?code=D07&showdescription=no) | D07 | 7527 | 27.9 |
| [Corticosteroids, dermatological preparations](https://www.whocc.no/atc_ddd_index/?code=D07&showdescription=no) | D07 | 1030 | 28.4 | [Drugs for constipation](https://www.whocc.no/atc_ddd_index/?code=A06&showdescription=no) | A06 | 7377 | 27.3 |

| **Supplementary table 4: List of ICD-10 codes used to extract data on the history of selected diseases pre-baseline (2015-2019)** | |
| --- | --- |
|  | **ICD-10** |
| **Cardiovascular diseases** | I3-I4, I50-I52, I6-I8, I10, I20-I28 |
| Diseases of veins. lymphatic vessels and lymph nodes. not elsewhere classified | I8 |
| Congestive heart failure | I50, I11-I15, I34-I35, I43-I47, I099, I420, I425-I429 |
| Atrial fibrillation | I48 |
| Essential hypertension | I10 |
| Stroke/transient ischemic attack | G45-G46, I63-I66, I693, I694 |
| Ischemic heart diseases | I20- I25 |
| Cerebrovascular diseases | I6 |
| Diseases of arteries. arterioles and capillaries | I7 |
| Peripheral arterial disease | I70-I71 I74, K55, I739 |
| Pulmonary heart disease and diseases of pulmonary circulation | I26-I28 |
| Myocardial infarction | I21-I23, I241, I252 |
| Angina pectoris | I20 |
| **Diabetes** |  |
| Diabetes type 2 | E11 |
| **Respiratory system** |  |
| COPD/Asthma | J43-J45 |
| **Musculoskeletal system** |  |
| Arthrosis | M15- M19 |
| Inflammatory polyarthropathies | M05-M14 |
| Systemic connective tissue disorders | M30-M36 |
| Infectious arthropathies | M00-M03 |


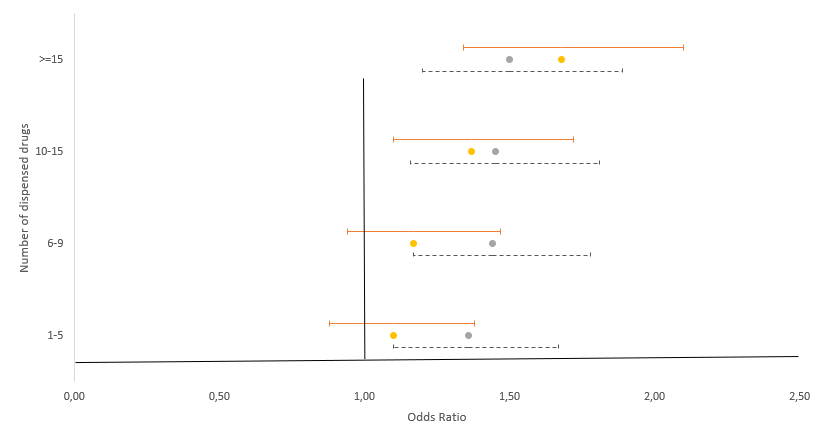


Supplementary Figure 1: Adjusted Odds ratios and 95% CI of COVID-19 infection associated with history of number of drug utilization among with premenopausal (Grey spot) or postmenopausal (Orange spot) breast cancer
